# The Molecular Basis and Biomarker Potential of the HIF-1α–VEGF–GLUT1 Hypoxia Axis in Laryngeal Squamous Cell Carcinoma

**DOI:** 10.3390/biomedicines14092103

**Published:** 2026-09-18

**Authors:** Wojciech Domka, Maciej Misiołek, Daniel Roshan Justin Raj, Dorota Bartusik-Aebisher, Angelika Myśliwiec, David Aebisher

**Affiliations:** 1 Department of Otolaryngology, Faculty of Medicine, Collegium Medicum, University of Rzeszów, 35-959 Rzeszów, Poland; 2 Department of Otorhinolaryngology and Oncological Laryngology in Zabrze, Medical University of Silesia, 40-055 Katowice, Poland; 3 English Division Science Club, Medical College of The Rzeszów University, 35-310 Rzeszów, Poland; dj135639@stud.ur.edu.pl; 4 Department of Biochemistry and General Chemistry, Faculty of Medicine, Collegium Medicum, University of Rzeszów, 35-959 Rzeszów, Poland; dbartusikaebisher@ur.edu.pl (D.B.-A.); amysliwiec@ur.edu.pl (A.M.); 5 Department of Photomedicine and Physical Chemistry, Faculty of Medicine, Collegium Medicum, University of Rzeszów, 35-959 Rzeszów, Poland

**Keywords:** laryngeal squamous cell carcinoma, tumour hypoxia, HIF-1α, vascular endothelial growth factor, glucose transporter 1, serum biomarkers, angiogenesis, metabolic reprogramming, treatment resistance, biomarker panel

## Abstract

In laryngeal squamous cell carcinoma, hypoxia plays a major role in growth, spread, and treatment resistance. HIF-1α, VEGF, and GLUT1 are three linked biomarkers that show different aspects of this response. Low oxygen can be sensed by HIF-1α, and it follows up by activating genes that help with the survival of the tumour cells. VEGF helps to form new blood vessels, while GLUT1 increases the uptake of glucose and maintains the production of energy even when there is a lack of oxygen. Tissue and serum studies have reported higher levels of these biomarkers in malignant than in benign or normal samples, together with associations with adverse clinicopathological features. However, their diagnostic and prognostic utility remains exploratory because clinically validated thresholds, diagnostic performance measures, and independent prospective validation remain limited. Combined assessment of these biomarkers may provide complementary information on hypoxic signalling, angiogenesis and metabolic adaptation, although its diagnostic or prognostic superiority over individual biomarkers has not yet been established. However, serum measurements can be influenced by pre-analytical and systemic factors. So, in order for these biomarkers to advance enough to be used in clinical laryngology, large multicenter studies and a standardized set of testing methods will be necessary.

## 1. Introduction

Laryngeal diseases can range from benign vocal fold lesions such as nodules, polyps, and papillomas, to malignancies, most importantly invasive laryngeal squamous cell carcinoma (LSCC). LSCC is a key environment for hypoxia-related biomarkers, as it affects swallowing, voice, and airway protection mechanisms. As such, the chosen treatment must provide adequate oncological control while preserving laryngeal functions. Laryngeal cancer is one of the most important malignancies of the upper aerodigestive tract, with squamous cell carcinoma accounting for almost 95% of laryngeal tumours [1]. Current diagnostic procedures still rely mainly on symptoms, endoscopy, imaging, histopathology, and tumour, node, metastasis (TNM) staging. However, these methods cannot fully apprehend tumour biology or provide predictions as to which lesions could behave aggressively. This is why molecular biomarkers are being explored as possible tools to provide earlier detection, risk stratification, and better prognosis in LSCC [2]. Benign laryngeal lesions are important comparison groups as they help with differentiating cancer-related molecular changes from inflammation, trauma, or epithelial hyperplasia. It has also been reported in serum studies that high concentrations of hypoxia-inducible factor 1-alpha (HIF-1α/HIF1A), glucose transporter type 1 (GLUT1), and vascular endothelial growth factor (VEGF) may be suggestive of cancer when diagnosing patients with laryngeal lesions [3]. Hypoxia is important as it is not just insufficient oxygen but also a biological signal that influences the behaviour of tumours. In solid tumours, the lack of oxygen stabilizes HIF-1α, which is known to regulate genes that are involved in adaptation, angiogenesis, and metabolism [4]. HIF-1α has also been linked with GLUT1 and VEGF in LSCC, which connects hypoxia with the uptake of glucose, glycolytic metabolism, and angiogenesis [5]. Another study on laryngeal carcinoma concluded that HIF-1α may stimulate GLUT1 and VEGF expression, which can in turn support tumour angiogenesis, invasion, and metastasis [6]. Clinically, this matters as hypoxic head and neck cancers are often aggressive and show resistance to radiotherapy or chemotherapy [7]. In head and neck squamous cell carcinoma (HNSCC), hypoxia can be observed to increase radioresistance. This makes oxygen-related markers even more important not only in diagnosis, but also in predicting the response to treatment [8].

**Figure 1:**
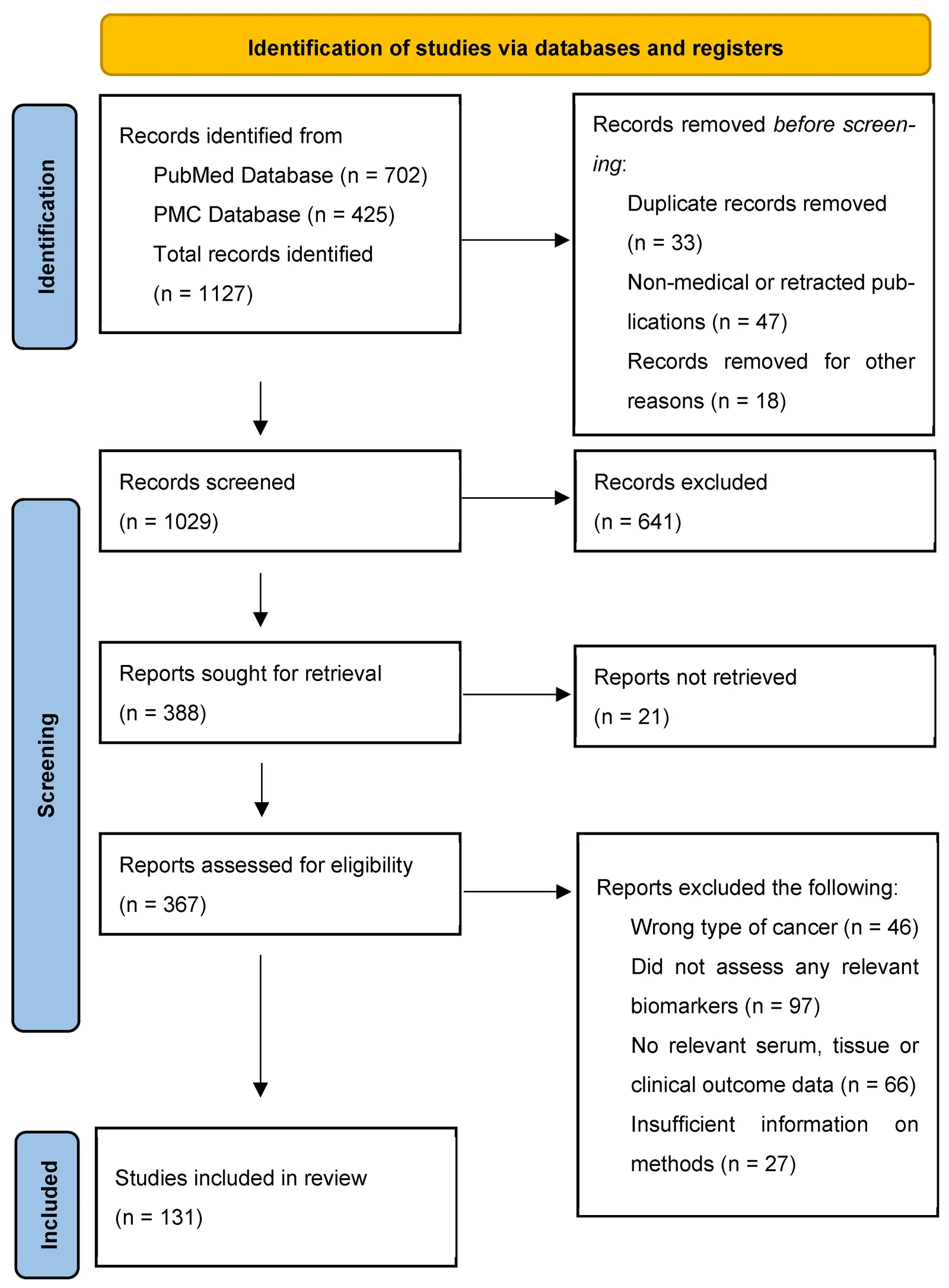
Preferred Reporting Items for Systematic Reviews and Meta-Analyses (PRISMA) flow diagram that represents the methods behind the selection of literature included in the review.

This review discusses serum HIF-1α, VEGF, and GLUT1 and their use as hypoxia-related biomarkers in laryngology, particularly in laryngeal squamous cell carcinoma. These three molecules were selected as they represent an interlinked aspect of the hypoxic tumour response. HIF-1α acts as a central transcription factor that is sensitive to oxygen. VEGF shows angiogenic signalling that is caused by hypoxia, while GLUT1 is an indicator of metabolic adaptation through increased glucose transport and glycolysis. It has been reported that serum and tissue concentrations of HIF-1α, VEGF, and GLUT1 have been higher in laryngeal carcinoma than in benign laryngeal lesions and controls. These findings support their potential usefulness during diagnosis [3]. Also discussed in this review are tissue-based studies that link HIF-1α and GLUT1 expression with tumour behaviour and prognosis [5]. Material suggesting that HIF-1α may regulate GLUT1 and VEGF so that it can promote angiogenesis, invasion, and metastasis [6] is also reviewed. Overall, this review combines biochemical mechanisms, methods of detection, and clinical relevance to determine whether these biomarkers can improve diagnosis and therapeutic stratification in laryngeal disease. A structured literature search was performed, with the study identification and selection process documented using a PRISMA flow diagram (Figure 1). A comprehensive search of the PubMed and PubMed Central (PMC) databases provided 1127 records. After the removal of duplicates (n = 33), non-medical or retracted publications (n = 47), and other reasons (n = 18), 1029 records were further screened. Following abstract screening, 388 reports were sought for full-text retrieval, out of which 367 were assessed for eligibility. After the exclusion of studies due to an unrelated cancer type (n = 46), lack of assessment of relevant biomarkers (n = 97), lack of relevant serum, tissue, or clinical outcome data (n = 66), and insufficient methodological information (n = 27), 131 studies were included in the final review.

## 2. Materials and Methods

### 2.1. Literature Search Strategy

A structured literature search was performed in PubMed and PubMed Central (PMC) from database inception to 30 August 2026. The search was designed to identify studies investigating the HIF-1α–VEGF–GLUT1 hypoxia axis and its individual components in laryngeal carcinoma, with particular emphasis on laryngeal squamous cell carcinoma (LSCC). In PubMed, searches combined “laryngeal carcinoma” or “laryngeal squamous cell carcinoma” with terms relating to “hypoxia”, “HIF-1 alpha”, “VEGF”, and “GLUT1”. Additional targeted PubMed searches addressing serum biomarkers, prognostic significance, radiosensitivity, and circulating hypoxia biomarkers in head and neck cancer were performed during revision. These searches contributed 15 of the 131 publications included in the final review. In PMC, searches combined “laryngeal carcinoma” with “hypoxia”, “HIF-1 alpha”, “VEGF”, or “GLUT1”. Database-specific automatic term mapping expanded these queries to relevant indexed terms and synonyms. The complete database-generated search strategies and applied limits are provided in Supplementary Table S1.

### 2.2. Eligibility Criteria and Study Selection

Studies were considered eligible if they provided evidence relevant to the HIF-1α–VEGF–GLUT1 hypoxia axis in laryngeal carcinoma or contributed to its biological, clinical, prognostic, therapeutic, or methodological interpretation. Eligible publications included clinical and translational studies of laryngeal carcinoma or head and neck cancer. Experimental studies investigating hypoxia, HIF-1α, VEGF, GLUT1, angiogenesis, glucose metabolism, treatment response, or related signalling mechanisms were also included. Foundational mechanistic studies describing the regulation and function of these pathways were included when they provided information directly relevant to the biological interpretation of the HIF-1α–VEGF–GLUT1 axis. Studies addressing circulating or tissue biomarkers and their measurement, as well as relevant review articles, were also eligible.

Evidence from other tumour types or experimental systems was included when it provided mechanistic or methodological information directly relevant to interpretation of the HIF-1α–VEGF–GLUT1 axis.

Publications were excluded if they did not provide information relevant to hypoxia, HIF signalling, VEGF-mediated angiogenesis, GLUT1-associated metabolism, laryngeal or head and neck cancer biology, biomarker assessment, or the clinical interpretation of these pathways. Duplicate publications and records that did not provide sufficient methodological or outcome information to determine eligibility or interpret the reported findings were also excluded. Titles and abstracts of retrieved records were screened for relevance, followed by full-text assessment of potentially eligible publications according to these criteria.

## 3. Biochemical Basis of Hypoxia Signalling

### 3.1. Oxygen Sensing and HIF-1α Regulation

**Figure 2:**
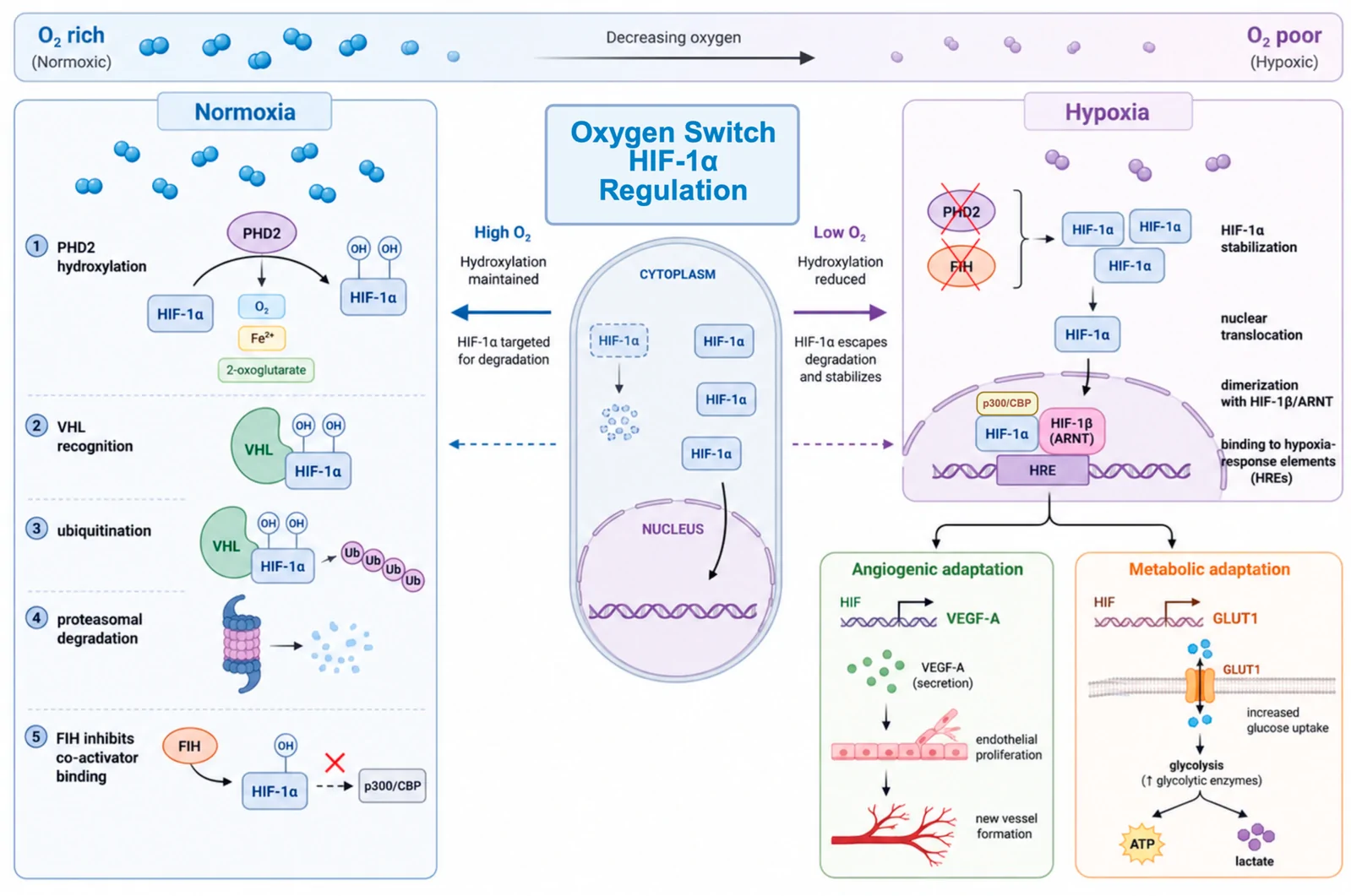
The oxygen-dependent regulation of HIF-1α and activation of angiogenic and metabolic responses. Under normoxic conditions, PHD enzymes hydroxylate HIF-1α, enabling its recognition by VHL and subsequent ubiquitination and proteasomal degradation. FIH independently hydroxylates HIF-1α and limits co-activator recruitment, further suppressing HIF-1 transcriptional activity. During hypoxia, reduced PHD activity allows HIF-1α to escape degradation and accumulate, while reduced FIH activity permits co-activator recruitment. HIF-1α then translocates to the nucleus, dimerises with HIF-1β/ARNT, and binds hypoxia-response elements. This activates downstream genes including VEGF, promoting angiogenesis, and GLUT1, increasing glucose uptake and glycolytic metabolism. Created in BioRender. Roshan, D. (2026). https://BioRender.com/e626kdg, accessed on 12 September 2026.

HIF-1 is an oxygen-responsive heterodimeric transcription factor composed of the oxygen-sensitive HIF-1α subunit and constitutively expressed HIF-1β/ARNT [9,10,11]. The oxygen-dependent degradation domain (ODD) of HIF-1α mediates its oxygen-dependent stability [12]. Under normoxic conditions, prolyl hydroxylase domain enzymes hydroxylate conserved proline residues within HIF-1α, enabling recognition by the von Hippel–Lindau tumour suppressor protein and subsequent ubiquitination and proteasomal degradation [13,14,15,16,17,18]. FIH-1 provides an additional oxygen-sensitive checkpoint by limiting co-activator recruitment [19,20]. During hypoxia, reduced hydroxylation allows HIF-1α to escape degradation, accumulate, and translocate to the nucleus, where it dimerises with HIF-1β/ARNT and activates hypoxia-response elements [21]. Among its downstream effects are increased VEGF-mediated angiogenesis and GLUT1-dependent glucose uptake, providing the mechanistic basis for considering these three molecules as complementary biomarkers of the hypoxic response, as seen in Figure 2. Importantly, these downstream markers should not be regarded as interchangeable measures of hypoxia, as HIF-1α reflects activation of oxygen-sensitive transcriptional signalling, whereas VEGF and GLUT1 represent distinct angiogenic and metabolic consequences of that response.

### 3.2. Transcriptional Responses Caused by Hypoxia

Following hypoxic stabilization, HIF-1 activates a broader transcriptional program that coordinates angiogenic, metabolic, and survival responses [11,22]. Oxygen delivery can be improved through this transcriptional program as it activates angiogenic genes, particularly VEGFA, whose promoter is stimulated by HIF-1 during hypoxia [23,24]. VEGF then goes on to stimulate endothelial proliferation, vascular permeability, and the formation of new vessels, which help hypoxic tissues have an increased blood supply. HIF-1α can also modify metabolism in such a way that allows cells to generate ATP with less oxygen. It can increase the uptake of glucose by promoting GLUT1, or solute carrier family 2 member 1 (SLC2A1). Glycolytic flux can be further improved by HIF-1α through the activation of genes that encode glycolytic enzymes such as aldolase, phosphoglycerate kinase, and lactate dehydrogenase A [25,26]. HIF-1 is also known to stimulate pyruvate dehydrogenase kinase 1 (PDK1), which leads to the inhibition of pyruvate dehydrogenase. This leads to the reduced entry of pyruvate into the tricarboxylic acid (TCA) cycle, leading to the production of lactate [27,28]. Hypoxia-driven transcription further helps with survival and invasion. The expression of HIF-1-dependent BCL2/adenovirus E1B 19kDa interacting protein 3 (BNIP3) and NIX can stimulate mitophagy and autophagy. This allows for the removal of damaged mitochondria due to prolonged hypoxia [29,30]. When it comes to tumours, HIF-1α can be seen to support invasion as it can regulate the remodelling of the extracellular matrix and metastatic signalling genes such as the LOX-family enzymes and CXCR4 [31,32,33].

### 3.3. Hypoxia, Angiogenesis and Metabolic Reprogramming

The two biggest consequential effects of hypoxia are angiogenesis and metabolic reprogramming. When HIF-1α is in a stabilized state, it activates genes that are responsive to hypoxia, which help cells restore oxygen delivery while also maintaining the production of ATP in low-oxygen conditions. HIF-1 under hypoxia induces the transcription of a key angiogenic target, vascular endothelial growth factor A (VEGF-A) [23]. This forms a link between oxygen sensing and vascular growth as the HIF system combines angiogenic signalling with the local oxygen demand [34]. VEGF plays an important role in supporting endothelial cell proliferation, migration, and vessel permeability, while HIF-1 is seen as the stimulator of VEGF-driven angiogenesis in both physiological and pathological conditions [34,35]. A response like this in tumours could potentially support growth, invasion, and treatment resistance as it creates new and abnormal vasculature [36]. At the same time, the presence of hypoxia turns metabolism from oxidative phosphorylation that is dependent on oxygen, towards glycolysis and the uptake of glucose [26,37]. GLUT1, which is encoded by SLC2A1, is particularly important as it can increase the amount of glucose entering hypoxic cells. Experimental studies have shown that hypoxia upregulates the expression of GLUT1 and glucose transport through mechanisms that are dependent on HIF-1 [38,39,40]. The expression of several glycolytic targets is also promoted by HIF-1α, which supports the generation of ATP during times when oxygen is limited [41,42]. Thus, angiogenesis mediated by VEGF and glucose metabolism mediated by GLUT1 are two representatives of complementary biochemical outputs of hypoxia signalling.

## 4. The Molecular Functions and Biomarker Roles of HIF-1α, VEGF, and GLUT1

### 4.1. HIF-1α: The Oxygen-Sensitive Master Regulator

The oxygen-sensitive subunit of HIF-1 is HIF-1α, which acts as a molecular switch between oxygen availability and adaptive gene expression. Initially, HIF-1 was identified as a hypoxia-induced DNA-binding factor at the erythropoietin enhancer [9]. Structurally, HIF-1 belongs to the basic helix-loop-helix (bHLH)–PER-ARNT-SIM (PAS) family, with domains responsible for DNA binding, dimerisation, and transcriptional activation [43,44]. As described in Section 3.1, HIF-1α abundance is tightly controlled by oxygen-dependent hydroxylation and VHL-mediated degradation, whereas hypoxia permits its stabilization and transcriptional activation [16,45,46]. Once HIF-1α has been stabilized, it dimerizes with HIF-1β and binds hypoxia-response elements in the target genes. This particularly activates the VEGF angiogenic program, which connects the lack of oxygen with vascular growth [23,47]. Metabolic adaptation is also supported by HIF-1α, as it induces the genes involved in the transport of glucose and glycolysis. Some of these include GLUT1, aldolase, enolase, and lactate dehydrogenase A, which ultimately helps with the production of ATP even when there is a lack of oxidative phosphorylation [22,48].

The expression of tissue HIF-1α in laryngeal carcinoma has been related to GLUT1, VEGF, tumour progression, and nodal metastasis. This supports that it has a role as a hypoxia-related tissue biomarker [49,50]. However, serum HIF-1α must be discussed more carefully. HIF-1α is mainly an intracellular transcription factor, unlike VEGF, which is a classical secreted protein. Studies that have measured circulating HIF proteins in laryngeal neoplasms have suggested that their levels may carry possible diagnostic or prognostic significance [3,51]. However, serum positivity should be taken as an indirect representation of tumour hypoxia, cell turnover or systemic disease biology, rather than as a direct piece of evidence pointing to active nuclear HIF-1α signalling. This differentiation is important in the interpretation of biomarkers, as tissue staining shows cellular localization, whereas in serum measurement, there is a loss of spatial context. This means that it cannot identify which specific tumour compartment produced the signal. For these reasons, although serum HIF-1α carries potential, it still requires validation against factors like tissue expression, tumour stage, and treatment response.

### 4.2. VEGF: The Angiogenic Hypoxia Marker

One of the most important molecular links between hypoxia and angiogenesis is VEGF, more particularly, VEGF-A. At first, it was considered a vascular permeability factor that tumour cells secreted. It was later characterized as a secreted, endothelial-specific angiogenic mitogen [52,53]. The expression of VEGF increases significantly in hypoxic tissues. Early research had shown that VEGF, which was hypoxia-induced, could regulate hypoxia-initiated angiogenesis [54]. This would mean that VEGF is activated after HIF signalling, as HIF-1 binds to the VEGF gene and increases its transcription during hypoxia [23]. Endothelial cells are mainly targeted by VEGF through the VEGF receptors, especially the vascular endothelial growth factor receptor 2 (VEGFR-2) or kinase insert domain receptor (KDR). This will activate the signalling pathways that control endothelial proliferation, permeability, and tube formation [55,56]. These processes are crucial for the formation of microvessels, as endothelial cells are pushed to proliferate, move to where there are angiogenic signals, and survive in new, developing vascular structures. As VEGF also increases vascular permeability, it allows plasma proteins to leak into the extracellular matrix. This will support the formation of a temporary stromal scaffold for vessel growth [57]. In tumours, this process is often abnormal. VEGF-driven angiogenesis leads to the formation of tortuous, leaky, and poorly organized vasculature, rather than producing mature vessels that are properly organized. Such outcomes can further worsen hypoxia and promote angiogenic signalling [58,59]. When it comes to laryngeal squamous cell carcinoma, VEGF is useful not only as an angiogenic regulator, but also as a biomarker of tumour vascularization. Immunohistochemical studies have observed VEGF in laryngeal carcinoma cells, stromal tissue, macrophages, and also in vascular endothelial cells. Strong VEGF staining can be related to an increase in microvessel density in some groups of patients [60,61]. The expression of VEGF has been linked with an overall decrease in the survival of patients with early-stage laryngeal cancer who were treated with radiotherapy. This supports the value of VEGF in prognosis [62]. Serum VEGF is a good example of a potential circulating biomarker, as VEGF is secreted, meaning it can be detected in blood, unlike HIF-1α and other intracellular transcription factors. Studies have reported a higher concentration of serum VEGF in patients with head and neck squamous cell carcinoma in comparison to healthy samples, which opens possibilities as a clinical monitoring tool [63]. In particular, the patients who were affected by advanced laryngeal carcinoma showed a level of serum VEGF that was significantly higher than that of healthy samples. Also, when patients with pharyngeal and laryngeal squamous cell carcinoma were treated with radiotherapy, changes were observed in the levels of serum VEGF [64,65]. Serum VEGF should therefore be interpreted cautiously, as circulating concentrations may reflect both tumour-related and systemic factors rather than tumour hypoxia alone.

### 4.3. GLUT1: The Metabolic Hypoxia Marker

**Figure 3:**
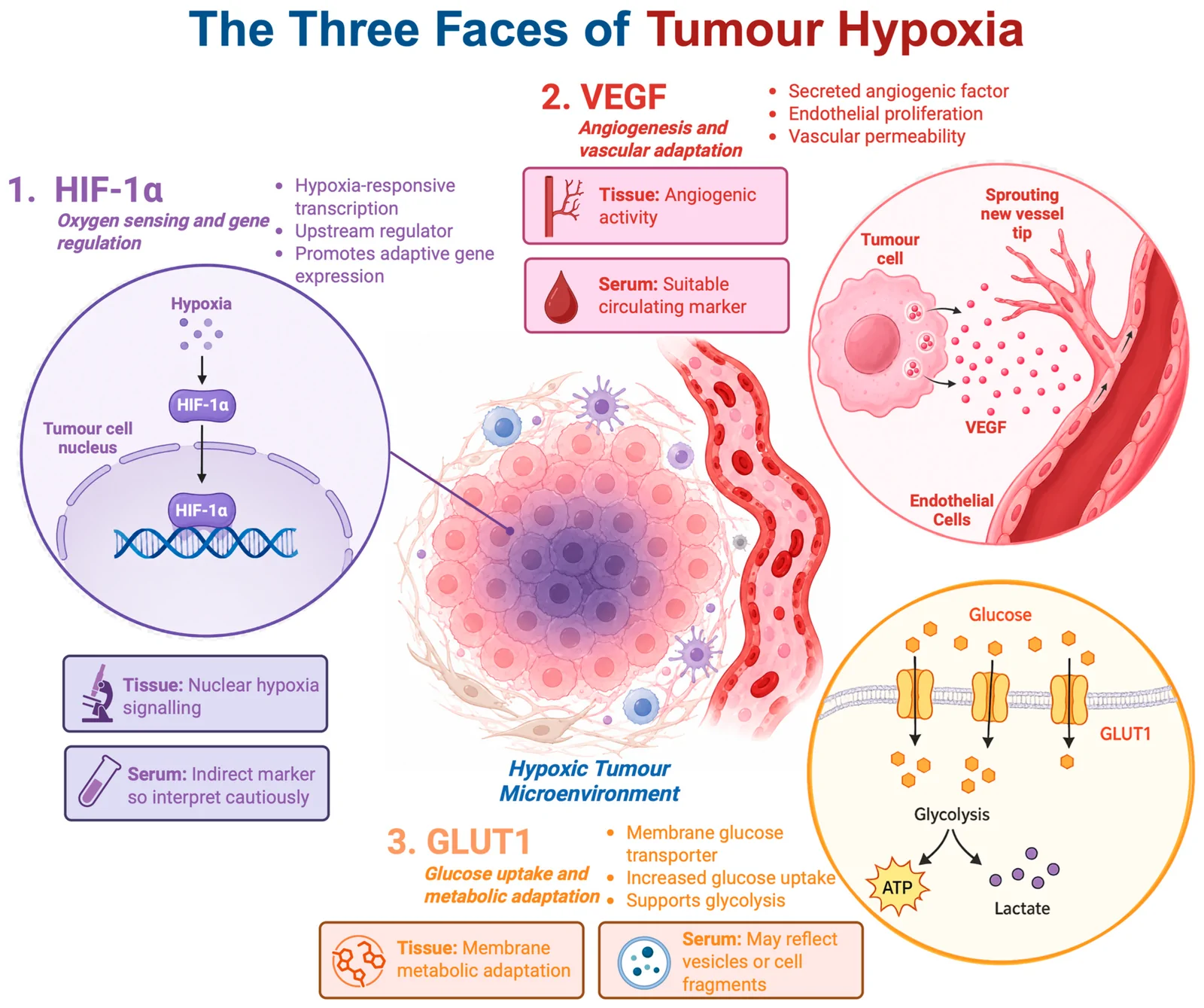
HIF-1α, VEGF, and GLUT1 are represented as the three faces of hypoxia. HIF-1α represents oxygen-sensitive transcriptional regulation, VEGF represents angiogenic and vascular adaptation, and GLUT1 represents increased glucose uptake and glycolytic metabolism. Their measurements from tissue and circulation give different but related information regarding the hypoxic tumour microenvironment. Created in BioRender. Roshan, D. (2026). https://BioRender.com/qn6pvsx, accessed on 12 September 2026.

GLUT1 is a facilitative glucose transporter encoded by SLC2A1 and located predominantly in the plasma membrane, where it mediates basal glucose uptake through energy-independent facilitated diffusion [66,67]. When there is a shortage of oxygen or nutrients, GLUT1 can increase in number and membrane localization, making sure that cells receive glucose despite there being a decrease in mitochondrial ATP production [40]. During hypoxic conditions, HIF-1α stabilizes and binds hypoxia-responsive regulatory sequences within the GLUT1 promoter and increases the transcription of SLC2A1 [39]. HIF-1 also promotes glycolytic metabolism, increasing glucose utilization and reducing reliance on oxygen-dependent mitochondrial metabolism. Increased glycolysis generates lactate and protons, which require effective export mechanisms to help maintain intracellular pH [68]. An increased expression of GLUT1 in tumours allows for more glucose to enter malignant cells. Through glycolysis, the glucose that entered is metabolized to produce pyruvate and lactate. Even in low oxygen, this helps to maintain the production of ATP while also providing intermediates that are required to synthesize nucleotides, amino acids, and lipids. Thus, GLUT1 plays a role in contributing to the Warburg effect, in which cancer cells have a high uptake of glucose and an increased production of lactate even when oxygen is readily available [69]. Aerobic glycolysis is known to generate lower amounts of ATP per glucose molecule when compared with oxidative phosphorylation. However, its faster rate of generation of intermediates helps in tumour-cell proliferation, redox homeostasis, and survival within the ever-changing tumour microenvironment [70]. Thus, the expression of GLUT1 is widely considered not as a direct measurement of oxygen concentration, but as a marker of metabolic adaptation, as illustrated in Figure 3 and summarized in Table 1. The staining of GLUT1 in tumour tissues is seen mostly in areas with low oxygen levels and shows the ability of cancer cells to take up more glucose. In several malignancies, the expression of GLUT1 correlated with measured tumour hypoxia and clinical complications [71]. GLUT1 has been connected with the expression of HIF-1α, tumour invasiveness, more advanced diseases, and poor prognosis. These observations support the idea that GLUT1 can be a metabolic-hypoxia biomarker [5,50,72]. However, serum GLUT1 requires substantially more cautious interpretation than tissue GLUT1. Unlike VEGF, GLUT1 is an integral membrane protein rather than a conventionally secreted soluble factor. The main available laryngeal carcinoma study reported higher serum GLUT1 concentrations in patients with cancer than in controls, alongside increased tissue concentrations [3]. However, this evidence was derived from a relatively small cohort of 52 patients, and an independent association between serum GLUT1 and tumour stage, prognosis or treatment response has not yet been established. The biological form represented by a serum GLUT1 measurement also remains uncertain. Circulating GLUT1 may be associated with extracellular vesicles or membrane fragments, while haemolysis and contamination by erythrocytes or other blood-derived material may contribute to the measured signal because GLUT1 is abundant in erythrocyte membranes. GLUT1 has also been identified in extracellular vesicles [73], supporting the biological plausibility of vesicle-associated circulating GLUT1, but this should not be considered equivalent to freely soluble GLUT1. Therefore, serum GLUT1 should currently be regarded as an exploratory biomarker rather than a clinically validated surrogate of tumour GLUT1 expression. Future studies should distinguish soluble, extracellular-vesicle-associated, and blood-cell-derived GLUT1, control for haemolysis and cellular contamination, and correlate circulating measurements with matched tumour GLUT1 expression and clinical outcomes.

## 5. The Integrated HIF-1α–VEGF–GLUT1 Axis in Laryngeal Carcinoma

### 5.1. The Integrated HIF-1α–VEGF–GLUT1 Response to Hypoxia

**Figure 4:**
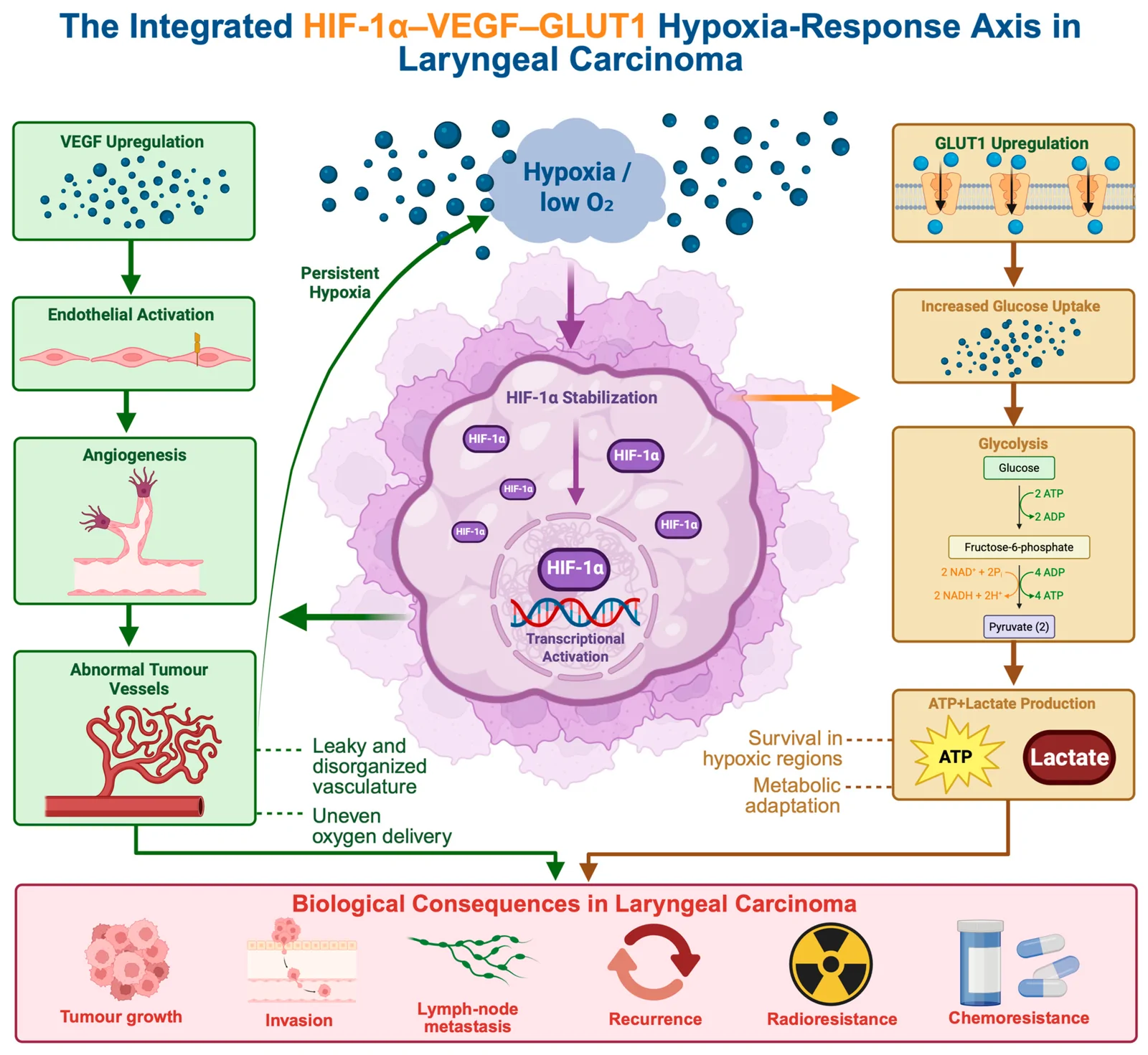
The integrated HIF-1α-VEGF-GLUT1 hypoxia-response axis in laryngeal carcinoma. Created in BioRender. Roshan, D. (2026). https://BioRender.com/hqzub6u, accessed on 12 September 2026.

As described above, HIF-1α is normally regulated through oxygen-dependent VHL-mediated proteasomal degradation, whereas tumour hypoxia permits its stabilization and activation [36,74,76]. In laryngeal carcinoma, downstream adaptive responses include VEGF-mediated angiogenesis and GLUT1-dependent metabolic reprogramming [77]. Laryngeal carcinoma models show this pathway very well. In human epithelial type 2 (HEp-2) cells, hypoxia increased HIF-1α protein alongside VEGF and GLUT1 expression [78]. This simultaneous increase demonstrates co-occurrence under hypoxic conditions but does not, by itself, establish a causal regulatory relationship between these proteins, as the study did not directly manipulate HIF-1α signalling or demonstrate downstream transcriptional dependence. Importantly, this co-occurring increase should not be interpreted as evidence of simultaneous or quantitatively equivalent regulation. HIF-1α acts upstream as the oxygen-sensitive transcriptional regulator, whereas VEGF and GLUT1 represent downstream transcriptional responses. HIF-1α stabilization therefore precedes the increased expression of these target proteins [21,23,39], although their subsequent kinetics and magnitude of induction may differ. Importantly, the available studies in laryngeal carcinoma have not provided directly comparable effect sizes that would allow numerical regulatory weights to be assigned to the HIF-1α–VEGF and HIF-1α–GLUT1 relationships. Their relative contributions may vary according to the severity and duration of hypoxia and additional tumour-specific signalling mechanisms. Positive associations between HIF-1α and VEGF [79] and also between HIF-1α, VEGF and GLUT1 [6] have also been found in other studies discussing laryngeal tumours. Likewise, an increased expression of HIF-1α and GLUT1 has been related to tumour invasiveness and prognosis [5]. Thus, VEGF and GLUT1 represent interconnected, rather than simply parallel, components of the hypoxic response. Their principal molecular connection is their shared upstream regulation by HIF-1α. However, additional cross-talk can occur at the level of the tumour vasculature. VEGF signalling can increase endothelial glucose metabolism, and experimental evidence has shown that VEGF can increase endothelial GLUT1 expression through the PI3K/Akt pathway, thereby coupling angiogenic stimulation with the increased glucose uptake required by proliferating endothelial cells [80]. This provides a functional link between VEGF-mediated angiogenesis and GLUT1-dependent metabolism. However, this VEGF–GLUT1 relationship has been demonstrated in endothelial models rather than specifically in laryngeal carcinoma and should therefore be regarded as mechanistically relevant cross-talk rather than an established tumour-cell-autonomous pathway in LSCC. VEGF has functions to stimulate the proliferation of endothelial cells and the formation of new vessels, helping the tumours receive more oxygen and nutrients [81]. However, oxygen deficiency may not be fully resolved as the tumour vessels are often malformed. A similar survival mechanism is provided by GLUT1, as it increases glucose uptake and prolongs glycolysis when there is a shortage of mitochondrial ATP production [82]. Proliferation and migration are reduced during hypoxic, low-glucose conditions in laryngeal carcinoma stem-like cells [83]. When HIF-1α and GLUT1 are inhibited together, tumour growth slows down, and radiosensitivity is improved in experimental laryngeal carcinoma [84,85]. Taken together, the mechanistic relationship between these molecules is hierarchical rather than equivalent. Hypoxia stabilizes HIF-1α, which transcriptionally promotes VEGF-mediated angiogenesis and GLUT1-mediated metabolic adaptation [21,23,39]. Therefore, VEGF and GLUT1 represent biologically distinct downstream outputs of HIF-1α signalling, while additional cross-talk between angiogenic and metabolic pathways may occur, particularly through VEGF-driven regulation of endothelial glucose metabolism. Their combined assessment may provide broader biological coverage of the hypoxic phenotype, but this mechanistic complementarity does not establish superior diagnostic or prognostic performance, as depicted in Figure 4.

### 5.2. Biological and Biomarker Relevance in Laryngeal Carcinoma

Laryngeal carcinoma can benefit from vascular and metabolic advantages provided by the HIF-1α–VEGF–GLUT1 axis as summarized in Table 2. HIF-1α supports the growth of tumours even in low-oxygen environments, as it promotes VEGF, which causes angiogenesis, and GLUT1, which increases the uptake of glucose. The expression of these molecules in laryngeal carcinoma tissues can be linked with angiogenesis, invasion, and metastasis [6]. The observation of HIF-1α and VEGF has also been correlated with advanced pathological tumour-node-metastasis (pTNM) stage and cervical lymph-node involvement [79]. The increased expression of VEGF and microvessel density has also been related to tumour invasion, metastasis, and recurrence [86]. Aggressive patterns of invasion can be observed with a high expression of GLUT1 and HIF-1α, while the epithelial to mesenchymal transition and the invasion of laryngeal cancer cells are stimulated by hypoxic conditions [87]. When there is an overexpression of HIF-1α and GLUT1 in tissues, often times it can be associated with recurrence, metastasis, and a reduction in survival [5]. A much shorter disease-free survival after surgery was seen in the HIF1A C1772T variant that is associated with high expression of tumour HIF-1α. Hypoxia has the ability to reduce the effectiveness of treatment, as the lack of oxygen reduces damage from radiation. However, HIF-1α signalling, abnormal vasculature due to VEGF, and GLUT1-supported glycolysis help cells to survive through the therapeutic stress. When the phosphoinositide 3-kinase/protein kinase B/mechanistic target of rapamycin (PI3K/Akt/mTOR) pathway was inhibited, HIF-1α and GLUT1 became suppressed, leading to better radiosensitivity in laryngeal carcinoma models [85]. In Hep-2 laryngeal carcinoma cells, suppression of both GLUT1 and phosphorylated Akt increased sensitivity to cisplatin, indicating that PI3K/Akt signalling contributes to chemoresistance alongside GLUT1 [88]. Measurements taken from tissues show a more accurate view of the local tumour biology, as they can provide expression within the malignant cells, invasive and hypoxic regions. Tissue sampling, however, is invasive and remains vulnerable to intratumoural spatial heterogeneity. Small biopsies interrogate only a limited tumour region, while even larger surgical specimens may not fully represent the entire lesion when biomarker assessment is performed on selected tissue blocks or regions. Serum assays, on the other hand, are less invasive and provide the opportunity for repeated monitoring. A laryngeal carcinoma study had found increased levels of HIF-1α, GLUT1 and VEGF in both tumour tissues and serum when compared with healthy samples [3]. However, circulating biomarker concentrations may also be influenced by pre-analytical and systemic factors and should therefore be interpreted alongside tissue findings [89,90]. Thus, serum measurements must be used together with tissue findings and have to be validated against the treatment response and recurrence.

### 5.3. Cross-Cancer Relevance of the HIF-1α–VEGF–GLUT1 Hypoxia Axis

The biological relevance of HIF-1α–VEGF–GLUT1 signalling is not restricted to laryngeal carcinoma. Similar hypoxia-adaptive responses have been reported in several other solid tumours, in which HIF-1α-associated signalling can support VEGF-mediated angiogenesis together with GLUT1-dependent glucose uptake and glycolytic metabolism. These complementary responses can provide tumour cells with an adaptive advantage under oxygen and nutrient limitation by supporting vascularisation, metabolic survival, tumour progression and, in some settings, treatment resistance. Representative examples are summarized in Table 3. However, evidence from other tumour types should be regarded as supporting the biological plausibility of the pathway rather than demonstrating that its diagnostic or prognostic performance can be directly extrapolated to LSCC.

## 6. The Clinical Application of the HIF-1α–VEGF–GLUT1 Axis in Laryngeal Carcinoma

### 6.1. Diagnostic and Prognostic Relevance

#### 6.1.1. Diagnostic Relevance

The diagnostic and prognostic utility of the HIF-1α–VEGF–GLUT1 axis in laryngeal carcinoma remains exploratory, although available studies have reported differences in biomarker expression between malignant and non-malignant tissues and associations with adverse clinical outcomes. In patients affected by laryngeal carcinoma, significantly higher concentrations of HIF-1α, VEGF, and GLUT1 were observed in serum and tumour tissues than in normal tissues or benign laryngeal lesions [3]. Importantly, benign laryngeal lesions represent a heterogeneous pathological group rather than a single biological entity. Some studies have examined individual non-malignant pathologies separately. For example, Wu et al. evaluated vocal cord polyps and vocal cord leukokeratosis independently and reported lower HIF-1α and GLUT1 expression than in laryngeal carcinoma [5]. However, comparable subtype-specific evidence remains limited, particularly for other benign lesions such as nodules and papillomas. Pooling different benign pathologies may therefore obscure subtype-specific differences in biomarker expression, and future diagnostic studies should stratify benign comparator groups according to histopathological diagnosis. A study comprising 120 LSCCs and 60 normal laryngeal tissues had similarly shown a higher expression of HIF-1α and VEGF in malignant samples [99]. When laryngeal tumours were compared with non-cancerous mucosa, the expression of HIF-1α and GLUT1 had been increased [72]. The expression of VEGF is much more frequently seen in LSCC than in the adjacent epithelium [100]. Measuring the biomarkers together may capture complementary hypoxic, angiogenic, and metabolic changes. However, superior diagnostic performance over individual biomarkers has not yet been established. However, sensitivity, specificity, clinically validated cut-off values, and independent prospective validation remain insufficient. These biomarkers should therefore currently be regarded as exploratory and, if assessed clinically, as complementary to rather than replacements for established endoscopic and histopathological evaluation.

#### 6.1.2. Prognostic Significance

In terms of prognosis, increased expression of components of the HIF-1α–VEGF–GLUT1 axis has been associated with several adverse clinical outcomes in LSCC, including lymph-node involvement, recurrence, metastasis and reduced survival. HIF-1α overexpression has been associated with lymph-node classification, recurrence, and metastasis, while GLUT1 expression correlated with recurrence and metastasis and was reported as an independent indicator of survival [5]. Increased HIF-1α and VEGF expression has also been associated with advanced TNM stage, cervical lymph-node metastasis, and reduced five-year overall survival [99]. Similarly, high VEGF expression has been linked with advanced stage, metastasis, and poorer survival [100], while VEGF-related angiogenesis has shown prognostic relevance in additional studies [60,101]. VEGF and CD31 were reported as prognostic biomarkers in early-stage LSCC [102], and VEGF-C expression and lymphatic vessel density were associated with nodal metastasis and regional recurrence [103]. CD105-based microvessel density has also been associated with lymph-node involvement and reduced overall survival [104]. In addition, the HIF1A C1772T variant was associated with poorer survival and increased risk of relapse [91]. Collectively, these findings suggest that increased hypoxic, angiogenic, and metabolic activity may identify biologically aggressive LSCC with a greater risk of recurrence, metastasis, or poorer survival. However, the prognostic evidence is heterogeneous, and these biomarkers have not yet been sufficiently validated to provide prognostic information independent of established clinicopathological factors such as TNM stage and nodal status. Standardized assays, predefined cut-offs, and prospective multivariable validation are therefore required before they can be incorporated into routine prognostic assessment [105].

### 6.2. Treatment-Modality-Specific Response and Combined Biomarker Assessment

#### 6.2.1. Radiotherapy Response

The effectiveness of radiotherapy is reduced significantly during hypoxia, as oxygen is needed to fix DNA damage caused by radiation. Importantly, HIF-1α, CA-IX and GLUT1 should not be regarded as equivalent indicators of the same oxygen tension, as their oxygen-response profiles differ. HIF-1 is a rapid oxygen-sensitive regulator whose DNA-binding activity and HIF-1α protein levels increase progressively as oxygen concentration decreases, with a half-maximal response reported at approximately 1.5–2% O_2_ and a maximal response at approximately 0.5% O_2_ [106]. CA-IX is a downstream hypoxia-responsive protein with a different oxygen dependency. In SiHa squamous carcinoma cells, maximal CA9 expression occurred at approximately 1% O_2_, and CA-IX protein followed the same expression pattern. GLUT1 also represents a downstream hypoxia-associated response but primarily reflects metabolic adaptation. In the same experimental model, maximal GLUT1 expression occurred at approximately 0.01% O_2_, substantially lower than the oxygen concentration producing maximal CA9 expression [107]. These differences were subsequently reproduced in both SiHa and FaDu pharyngeal squamous carcinoma cells, in which CA9 expression was maximal at 1% O_2_ whereas GLUT1 expression was maximal at 0.01% O_2_ [108]. These oxygen-response values are model-dependent rather than fixed clinical thresholds, but they demonstrate that HIF-1α, CA-IX and GLUT1 reflect overlapping rather than identical components of tumour hypoxia. Accordingly, these markers provide complementary rather than interchangeable information about radiobiologically relevant hypoxia.

The level of HIF-1α expression prior to treatment may be relevant to radiotherapy response. High expression of HIF-1α or CA-IX has been linked with residual tumour after radiotherapy [109]. Hypoxia-related proteins have also been reported as potentially relevant to local control, although methodological heterogeneity has limited their clinical implementation [110]. For GLUT1, experimental evidence is particularly strong. Suppression of GLUT1 increased radiation sensitivity and apoptosis in laryngeal carcinoma cells and xenografts [111], while inhibition of HIF-1α and GLUT1 reduced tumour growth, microvessel density and radioresistance [84]. Inhibition of PI3K/Akt/mTOR signalling similarly suppressed HIF-1α and GLUT1 and improved radiosensitivity in laryngeal carcinoma models [85].

Evidence for VEGF as a predictor of radiotherapy response is less consistent. VEGF expression did not differentiate between radiosensitive and radioresistant early laryngeal carcinoma models [112]. However, VEGF positivity was associated with poorer survival following primary radiotherapy [62], while serum VEGF concentrations were reported to decrease following irradiation [65]. Overall, HIF-1α and GLUT1 have stronger mechanistic evidence relating to radioresistance, whereas the predictive value of VEGF for radiotherapy response remains uncertain.

#### 6.2.2. Chemotherapy Response

Chemotherapy resistance in laryngeal carcinoma is multifactorial and is not restricted to GLUT1-dependent metabolic adaptation. PI3K/Akt signalling has been implicated specifically in cisplatin resistance. In Hep-2 cells, suppression of GLUT1 and phosphorylated Akt increased cisplatin sensitivity [88], while direct inhibition of PI3K/Akt with LY294002 acted synergistically with cisplatin to suppress tumour-cell proliferation [113]. AMPK signalling may provide an additional metabolic mechanism of resistance. In LSCC models, MNAT1/GDF15 signalling reduced AMPK phosphorylation and mitochondrial apoptosis, whereas MNAT1 or GDF15 knockdown increased cisplatin sensitivity, including in cisplatin-resistant cells and in vivo [114]. The WISP1/YAP1/TEAD1/GLUT1 pathway represents another mechanism through which WISP1-driven GLUT1 upregulation supports glycolysis and cisplatin resistance [115]. Collectively, these findings indicate that chemoresistance in LSCC involves interacting PI3K/Akt, AMPK-regulated mitochondrial and GLUT1-dependent metabolic pathways rather than a single WISP1–GLUT1 mechanism.

#### 6.2.3. Experimental Pathway-Targeted Approaches

The pathway-directed interventions described above should be distinguished from established targeted therapies in clinical LSCC. Experimental inhibition of HIF-1α, GLUT1 and PI3K/Akt/mTOR signalling has increased radiosensitivity in preclinical laryngeal carcinoma models [84,85], while inhibition of GLUT1- and PI3K/Akt-related signalling has increased cisplatin sensitivity [88,115]. The broader oncogenic relevance of this pathway is also supported by recent evidence in hepatocellular carcinoma, where LARS was shown to promote tumour progression through PI3K/AKT/mTOR signalling [116], as seen in Table 4. The laryngeal carcinoma studies therefore provide evidence for experimental treatment sensitisation rather than demonstrating a clinically validated response to targeted therapy.

#### 6.2.4. Combined Biomarker Assessment

The relevance of HIF-1α, VEGF and GLUT1 therefore differs according to treatment modality, as summarized in Table 4. HIF-1α and GLUT1 have been investigated predominantly in relation to radioresistance, while GLUT1 and interacting metabolic and pro-survival pathways have also been implicated in cisplatin resistance. Evidence concerning VEGF as a treatment-response marker is less consistent. Combined assessment of HIF-1α, VEGF and GLUT1 may capture complementary hypoxic, angiogenic and metabolic characteristics of the tumour. However, superior prediction of radiotherapy, chemotherapy or targeted-treatment response compared with individual biomarkers has not yet been established.

## 7. Analytical Challenges and Current Limitations

### 7.1. Sample Types and Detection Methods

Information provided by serum, plasma, and tissue about the HIF-1α–VEGF–GLUT1 axis is related but not identical to each other. Blood sampling is only minimally invasive, which makes serum or plasma a suitable candidate for larger clinical groups and repeated monitoring. Increased concentrations of HIF-1α, VEGF, and GLUT1 were seen in both serum and tumour tissue in laryngeal carcinoma patients [3]. However, circulating levels of these biomarkers may become influenced by systemic production and blood-cell release rather than just from the tumour alone. VEGF is particularly sensitive to the type of sample, as platelet activation during clotting can raise serum concentrations proportional to plasma [117,118]. Tissue specimens usually represent local tumour biology better and preserve cellular localization. However, sampling error is not limited to small biopsies; although biopsies interrogate only a restricted tumour region, larger surgical specimens may also incompletely represent spatially heterogeneous tumours when only selected blocks or regions are analyzed. Various detection methods can be used to examine different biological levels. Enzyme-linked immunosorbent assay (ELISA), for example, can give a quantitative measurement of soluble proteins, although its accuracy depends on certain factors like antibody specificity, calibration, and matrix effects [119]. Commercially available serum-compatible ELISA assays for these biomarkers show differing levels of documented analytical specificity. For HIF-1α, the Cloud-Clone human HIF-1α ELISA (SEA798Hu), which is validated for serum and plasma, is reported by the manufacturer to show no significant cross-reactivity or interference with HIF-1α analogues, although a quantitative cross-reactivity panel is not provided. For GLUT1, the Nori Human GLUT1/SLC2A1 ELISA (GR111153) reports < 0.5% cross-reactivity with available related molecules and no significant interference from the related molecules evaluated. More detailed quantitative data are available for some VEGF assays as well. For example, the Invitrogen Human VEGF ELISA (KHG0111) reports 0.25% and 0.11% cross-reactivity with mouse and rat VEGF165, respectively, while human VEGF121 shows 100% cross-reactivity with human VEGF165. These data demonstrate that analytical specificity is assay-dependent and that manufacturers differ considerably in the extent of cross-reactivity testing reported. Future serum studies should therefore report the manufacturer, catalogue number, isoform recognition, and cross-reactivity characteristics of the ELISA used. Immunohistochemistry gives a view of protein localization and staining intensity, but variability is a concern due to scoring and antibody validation [120]. Quantitative polymerase chain reaction (qPCR) can measure VEGFA and SLC2A1 transcripts, while Western blotting can reveal relative protein abundance and molecular size. Both of these methods have been previously applied in laryngeal carcinoma models and cell models [49,72]. Through multiplex assays, several biomarkers can be analyzed using small sample volumes. However, cross-reactivity and platform-specific differences call for the need for strong validation [121].

### 7.2. Sources of Variability and Gaps in Evidence

Pre-analytical conditions play a major role in the interpretation of HIF-1α, VEGF, and GLUT1 measurements. Many factors like delays prior to centrifugation, type of collection tube, storage temperature, and several freeze–thaw cycles can influence the measured concentration. This also reduces the comparability between different studies [122]. For VEGF, platelet release during clotting can increase serum concentrations, making sample type and standardized processing important when comparing circulating measurements [118,123]. Procedures like hemolysis can release intracellular proteins and cause assay interference, while a systemic infection or inflammation could increase the level of circulating VEGF with no relation to tumour activity [124]. As regional hypoxia, necrosis, vascularity and metabolism can vary within tumours, tissue-based biomarker measurements are vulnerable to spatial sampling error. This limitation is particularly important for small biopsies, but larger surgical specimens are also affected because biomarker expression may differ across tumour regions and routine analysis generally examines only selected sections or tissue blocks [104,125]. Multi-region sampling or standardized sampling of representative tumour areas may therefore provide a more complete assessment of intratumoural heterogeneity. An important unresolved issue is the cellular origin of circulating HIF-1α. Future validation studies should therefore use paired tumour and blood samples collected from the same patients and fractionate blood into cell-free, preferably platelet-poor plasma and cellular components. HIF-1α should be measured independently in tumour tissue, plasma, leukocyte, erythrocyte and platelet fractions, with haemolysis and residual platelet contamination documented as pre-analytical controls. Extracellular-vesicle-associated HIF-1α could additionally be assessed after enrichment for tumour-associated vesicle populations using appropriate epithelial or tumour markers. Longitudinal sampling before and after tumour resection or treatment would provide further evidence of origin, as a reduction in circulating HIF-1α paralleling tumour removal or response, without a corresponding change in blood-cell-associated HIF-1α, would support a tumour contribution. Such source-resolved studies are required before serum or plasma HIF-1α can be interpreted as a tumour-specific biomarker. The available literature on laryngeal carcinomas remains limited. The literature search was restricted to PubMed and PubMed Central, which may have resulted in the omission of relevant publications indexed exclusively in other bibliographic databases. This should be considered when interpreting the comprehensiveness of the evidence base. Some of the important studies have been conducted with only 52 patients [3] or 35 patients [6], which limits subgroup analyses and gives potentially unstable associations. Standardized diagnostic or prognostic cut-off values have not been established, with the differences in assays, antibodies, staining thresholds, and scoring procedures further hindering comparisons between different studies. The reporting recommendations for tumour marker prognostic studies (REMARK) guidelines put emphasis on having transparent methods, predefined analyses, and independent validation [126]. Thus, there is a need for larger studies that follow patients over time and confirm their results across various centres with consistent methods to fully consider these biomarkers for use in everyday clinical practice [127].

## 8. Future Perspectives and Conclusions

Future research should first validate HIF-1α, VEGF and GLUT1 as a biologically defined multi-marker panel reflecting hypoxic signalling, angiogenesis and metabolic adaptation. Other liquid-biopsy analytes, including circulating tumour DNA, circulating tumour cells and microRNAs, may provide complementary information on tumour burden and molecular alterations, but they should not be regarded as components of the HIF-1α–VEGF–GLUT1 axis in the absence of evidence demonstrating a direct mechanistic relationship with hypoxia signalling. Future studies could instead determine whether integrating these independent liquid-biopsy markers with hypoxia-related biomarkers provides additional diagnostic, prognostic or treatment-response information [128]. Extracellular vesicles may provide an additional platform for circulating biomarker assessment because they contain tumour-derived proteins, lipids and nucleic acids protected within membrane-bound particles. However, their isolation and analytical methods still require further standardization [129]. In particular, future studies should incorporate paired tumour–blood sampling, blood-cell fractionation and tumour-enriched extracellular-vesicle analysis to determine whether circulating HIF-1α originates predominantly from malignant tissue or from hematological and systemic sources. Rather than interpreting them separately, serum biomarkers should be integrated along with imaging. Studies in HNSCC have directly combined circulating hypoxia-related biomarkers with functional hypoxia imaging. Plasma biomarkers, including VEGF, have been assessed alongside [^18^F] FMISO PET/CT and [^18^F] HX4 PET/CT, although their correlations with imaging-defined hypoxia were variable [130,131]. These findings support further investigation of combined biomarker–imaging approaches, but their ability to guide biologically adapted radiotherapy remains unvalidated. Monitoring biomarkers and imaging before, during, and after the treatment can help doctors to tailor follow-ups and adjust treatment according to the patient’s needs. It can also help with identifying recurrence earlier, but these models will have to undergo rigorous multicenter testing and validation before they can be clinically implemented.

HIF-1α, VEGF and GLUT1 form a hypoxia-related axis that is biologically consistent in laryngeal carcinoma. HIF-1α represents oxygen sensing while VEGF represents angiogenic adaptation and GLUT1 represents metabolic adaptation. Together, these markers capture complementary aspects of hypoxia-related tumour biology, although their clinical superiority over individual biomarkers has not yet been demonstrated. Serum assessment of these biomarkers should therefore currently be regarded as exploratory rather than clinically validated for diagnosis, prognosis or monitoring. Standardized assays, predefined clinical cut-offs and large prospective multicentre studies comparing circulating measurements with matched tissue expression, imaging findings and clinical outcomes are required before routine clinical implementation.

## Figures and Tables

**Table 1:** The molecular functions and biomarker roles of HIF-1α, VEGF, and GLUT1.

Biomarker and Focus	Main Function	Relevance to Laryngeal Cancer	Biomarker Interpretation
Function of HIF-1α [74]	Activates the genes that are involved in hypoxia adaptation.	Regulates VEGF and GLUT1 expression.	Indicates the activation of hypoxia signalling.
HIF-1α as a biomarker [72]	Supports the survival of tumours and their progression.	Higher expression may be connected with aggressive disease.	Tissue measurement is preferred; serum results require caution.
Function of VEGF [75]	Promotes angiogenesis and vascular permeability.	Supports tumour blood-vessel formation.	Shows the angiogenic response to hypoxia.
VEGF as a biomarker [64]	Secreted into the tumour environment and blood.	Increased tissue or serum levels may indicate advanced disease.	Good for tissue and circulating measurement.
Function of GLUT1 [40]	Increases overall glucose uptake and glycolysis.	It helps tumour cells survive under hypoxia.	Depicts metabolic adaptation.
GLUT1 as a biomarker [72]	Shows increased tumour glucose use.	Associated with tumour invasion and progression.	Tissue measurement is more reliable than serum measurement.

**Table 2:** The integrated roles of the HIF-1α–VEGF–GLUT1 axis in laryngeal carcinoma.

Process	Main Molecules Involved	Biological Effect	Relevance to Laryngeal Carcinoma
Hypoxic activation [6]	HIF-1α, VEGF, GLUT1	Hypoxia is associated with increased HIF-1α, VEGF, and GLUT1 expression.	Shows co-occurring hypoxia-associated responses but does not establish causal regulation.
Angiogenesis [79]	HIF-1α and VEGF	Stimulates the formation of new blood vessels.	Associated with advanced stage and lymph-node involvement.
Metabolic adaptation [5]	HIF-1α and GLUT1	Increases glucose uptake and glycolysis.	Supports tumour survival in hypoxic regions.
Recurrence and prognosis [91]	HIF-1α	Increased hypoxia-related signalling.	May indicate a greater risk of recurrence.
Treatment resistance [85]	HIF-1α and GLUT1	Supports tumour growth and radioresistance.	Their suppression increases radiosensitivity.
Biomarker assessment [3]	HIF-1α, VEGF, GLUT1	Can be measured in tissue and in serum.	Higher concentrations may help distinguish carcinoma.

**Table 3:** Representative evidence for the biological relevance of HIF-1α–VEGF–GLUT1 signalling in other cancers.

Cancer Type	Evidence Involving the Pathway	Tumour-Promoting Advantage	Biological/Clinical Relevance
Gastric cancer	HIF-1α/HIF-1β signalling increased VEGF and hypoxia-responsive glucose-metabolism genes, including GLUT1, under hypoxic conditions [92].	Couples angiogenic adaptation with increased glucose utilization during hypoxia.	Supports survival and metabolic adaptation of gastric tumour cells in a hypoxic microenvironment.
Colorectal cancer	HIF-1α expression was associated with VEGF expression and microvessel density, while HIF-1α and GLUT1 showed linked expression in colorectal tumours [93,94].	Provides complementary vascular and glycolytic adaptation that can support tumour growth and invasion.	HIF-1α/VEGF signalling was associated with invasion and metastatic characteristics, while GLUT1 expression was greater in node-positive tumours in one study.
Pancreatic ductal adenocarcinoma	HIF-1α expression correlated positively with both VEGF and GLUT1, and increased expression of all three proteins was associated with advanced tumour stage and lymph-node metastasis [95].	Supports angiogenesis, glucose uptake, and survival within the markedly hypoxic pancreatic tumour microenvironment.	High HIF-1α, VEGF, and GLUT1 expression was associated with poorer overall survival.
Cervical cancer	HIF-1α, VEGF, and GLUT1 were concurrently evaluated as hypoxia-associated markers in cervical carcinoma [96].	Facilitates adaptation to tumour hypoxia and may contribute to resistance to radiation-induced damage.	Higher expression of hypoxia-associated markers was associated with poorer survival in stage III disease and greater radiotherapy-related resistance.
Hepatocellular carcinoma	HIF-1α expression was associated with VEGF and tumour microvessel density, while complementary experiments demonstrated HIF-1α-dependent induction of GLUT1 by hypoxia [97,98].	Combines increased tumour vascularisation with glycolytic adaptation and enhanced glucose uptake.	HIF-1α/VEGF signalling was linked with neovascularisation, while GLUT1 promoted HCC growth and migration.

**Table 4:** Potential clinical applications of the HIF-1α–VEGF–GLUT1 axis in laryngeal carcinoma.

Clinical Application	Biomarker	Main Finding	Clinical Interpretation
Diagnostic differentiation [3]	HIF-1α, VEGF and GLUT1	Tissue and serum concentrations were higher in laryngeal carcinoma than in benign lesions and healthy samples.	Higher concentrations were observed in malignant samples than in pooled benign or healthy samples; subtype-specific diagnostic performance and overall sensitivity and specificity remain unvalidated.
Tumour staging [99]	HIF-1α and VEGF	Higher expression was seen with advanced TNM stage and lymph-node metastasis.	Association with aggressive disease has been reported, but clinical staging utility remains unvalidated.
Prognostic assessment [5]	HIF-1α and GLUT1	Increased expression was connected with invasion, metastasis, and lower survival.	Associations with recurrence and survival have been reported, but prognostic utility remains exploratory and requires independent validation.
Radiotherapy response [84]	HIF-1α and GLUT1	Their simultaneous inhibition reduced tumour growth and increased radiosensitivity in experimental models.	Preclinical evidence links HIF-1α and GLUT1 with radioresistance; clinical predictive utility remains unvalidated.
Chemotherapy response [88,113,114,115]	GLUT1, PI3K/Akt and AMPK-related signalling	GLUT1 and PI3K/Akt inhibition increased cisplatin sensitivity, while WISP1–GLUT1 and MNAT1/GDF15–AMPK signalling were associated with resistance.	Chemoresistance reflects interacting metabolic and pro-survival pathways rather than GLUT1 alone.
Experimental pathway-targeted sensitisation [84,85,88,115]	HIF-1α, GLUT1 and PI3K/Akt/mTOR-related signalling	Pathway inhibition increased radio- or cisplatin sensitivity in experimental laryngeal carcinoma models.	Supports preclinical treatment-sensitizing strategies rather than a clinically validated targeted-therapy response biomarker.
Combined biomarker assessment [78]	HIF-1α, VEGF and GLUT1	All three proteins increased together under hypoxic conditions.	A combined panel may reflect hypoxic, angiogenic and metabolic changes, but requires clinical validation.

## Data Availability

No new data were created or analyzed in this study. Data sharing is not applicable to this article.

## Supplementary Materials

The following supporting information can be downloaded at: https://www.mdpi.com/article/10.3390/biomedicines14092103/s1, Table S1: Database-specific search strategies.
