# Supplementary material for: The Molecular Basis and Biomarker Potential of the HIF-1α–VEGF–GLUT1 Hypoxia Axis in Laryngeal Squamous Cell Carcinoma
Source: Biomedicines. 2026 Sep 18;14(9):2103. doi: 10.3390/biomedicines14092103 (PMC13604895; doi:10.3390/biomedicines14092103)
Supplement: Supplementary file 1 [file biomedicines-14-02103-s001.zip › biomedicines-4478560-supplementary.pdf]

Supplementary Materials

Supplementary Table S1. Database-specific search strategies.

Database	Search strategy
PubMed	**Search: laryngeal carcinoma HIF-1 alpha** ("larynges"[All Fields] OR "laryngitis"[MeSH Terms] OR "laryngitis"[All Fields] OR "laryngitides"[All Fields] OR "larynx"[MeSH Terms] OR "larynx"[All Fields] OR "laryngeal"[All Fields]) AND ("carcinoma"[MeSH Terms] OR "carcinoma"[All Fields] OR "carcinomas"[All Fields] OR "carcinoma s"[All Fields]) AND ("hif1a protein human"[Supplementary Concept] OR "hif1a protein human"[All Fields] OR "hif 1 alpha"[All Fields]) **Filters/limits: None**
PubMed	**Search: laryngeal carcinoma VEGF** ("larynges"[All Fields] OR "laryngitis"[MeSH Terms] OR "laryngitis"[All Fields] OR "laryngitides"[All Fields] OR "larynx"[MeSH Terms] OR "larynx"[All Fields] OR "laryngeal"[All Fields]) AND ("carcinoma"[MeSH Terms] OR "carcinoma"[All Fields] OR "carcinomas"[All Fields] OR "carcinoma s"[All Fields]) AND ("vascular endothelial growth factor a"[Supplementary Concept] OR "vascular endothelial growth factor a"[All Fields] OR "vegf"[All Fields] OR "vascular endothelial growth factor a"[MeSH Terms]) **Filters/limits: None**
PubMed	**Search: laryngeal carcinoma GLUT1** ("larynges"[All Fields] OR "laryngitis"[MeSH Terms] OR "laryngitis"[All Fields] OR "laryngitides"[All Fields] OR "larynx"[MeSH Terms] OR "larynx"[All Fields] OR "laryngeal"[All Fields]) AND ("carcinoma"[MeSH Terms] OR "carcinoma"[All Fields] OR "carcinomas"[All Fields] OR "carcinoma s"[All Fields]) AND "GLUT1"[All Fields] **Filters/limits: None**
PubMed	**Search: laryngeal squamous cell carcinoma HIF-1 alpha** ("squamous cell carcinoma of head and neck"[MeSH Terms] OR ("squamous"[All Fields] AND "cell"[All Fields] AND "carcinoma"[All Fields] AND "head"[All Fields] AND "neck"[All Fields]) OR "squamous cell carcinoma of head and neck"[All Fields] OR ("laryngeal"[All Fields] AND "squamous"[All Fields] AND "cell"[All Fields] AND "carcinoma"[All Fields]) OR "laryngeal squamous cell carcinoma"[All Fields]) AND ("hif1a protein human"[Supplementary Concept] OR "hif1a protein human"[All Fields] OR "hif 1 alpha"[All Fields]) **Filters/limits: None**
PubMed	**Search: laryngeal squamous cell carcinoma VEGF** ("squamous cell carcinoma of head and neck"[MeSH Terms] OR ("squamous"[All Fields] AND "cell"[All Fields] AND "carcinoma"[All Fields] AND "head"[All Fields] AND "neck"[All Fields]) OR "squamous cell carcinoma of head and neck"[All Fields] OR ("laryngeal"[All Fields] AND "squamous"[All Fields] AND "cell"[All Fields] AND "carcinoma"[All Fields]) OR "laryngeal squamous cell carcinoma"[All Fields]) AND ("vascular endothelial growth factor a"[Supplementary Concept] OR "vascular endothelial growth factor a"[All Fields] OR "vegf"[All Fields] OR "vascular endothelial growth factor a"[MeSH Terms]) **Filters/limits: None**
PubMed	**Search: laryngeal squamous cell carcinoma GLUT1** ("squamous cell carcinoma of head and neck"[MeSH Terms] OR ("squamous"[All Fields] AND "cell"[All Fields] AND "carcinoma"[All Fields] AND "head"[All Fields] AND "neck"[All Fields]) OR "squamous cell carcinoma of head and neck"[All Fields] OR ("laryngeal"[All Fields] AND "squamous"[All Fields] AND "cell"[All Fields] AND "carcinoma"[All Fields]) OR "laryngeal squamous cell carcinoma"[All Fields]) AND "GLUT1"[All Fields] **Filters/limits: None**

Supplementary Materials

PubMed	**Search: laryngeal carcinoma hypoxia** ("larynges"[All Fields] OR "laryngitis"[MeSH Terms] OR "laryngitis"[All Fields] OR "laryngitides"[All Fields] OR "larynx"[MeSH Terms] OR "larynx"[All Fields] OR "laryngeal"[All Fields]) AND ("carcinoma"[MeSH Terms] OR "carcinoma"[All Fields] OR "carcinomas"[All Fields] OR "carcinoma s"[All Fields]) AND ("hypoxia"[MeSH Terms] OR "hypoxia"[All Fields] OR "hypoxia s"[All Fields] OR "hypoxias"[All Fields]) **Filters/limits: None**
PubMed	**Search: laryngeal carcinoma serum biomarkers** ("larynges"[All Fields] OR "laryngitis"[MeSH Terms] OR "laryngitis"[All Fields] OR "laryngitides"[All Fields] OR "larynx"[MeSH Terms] OR "larynx"[All Fields] OR "laryngeal"[All Fields]) AND ("carcinoma"[MeSH Terms] OR "carcinoma"[All Fields] OR "carcinomas"[All Fields] OR "carcinoma s"[All Fields]) AND ("serum"[MeSH Terms] OR "serum"[All Fields] OR "serums"[All Fields] OR "serum s"[All Fields] OR "serumal"[All Fields]) AND ("biomarker s"[All Fields] OR "biomarkers"[Supplementary Concept] OR "biomarkers"[All Fields] OR "biomarker"[All Fields] OR "biomarkers"[MeSH Terms]) **Filters/limits: None**
PubMed	**Search: laryngeal carcinoma VEGF prognosis** ("larynges"[All Fields] OR "laryngitis"[MeSH Terms] OR "laryngitis"[All Fields] OR "laryngitides"[All Fields] OR "larynx"[MeSH Terms] OR "larynx"[All Fields] OR "laryngeal"[All Fields]) AND ("carcinoma"[MeSH Terms] OR "carcinoma"[All Fields] OR "carcinomas"[All Fields] OR "carcinoma s"[All Fields]) AND ("vascular endothelial growth factor a"[Supplementary Concept] OR "vascular endothelial growth factor a"[All Fields] OR "vegf"[All Fields] OR "vascular endothelial growth factor a"[MeSH Terms]) AND ("prognosis"[MeSH Terms] OR "prognosis"[All Fields] OR "prognoses"[All Fields]) **Filters/limits: None**
PubMed	**Search: laryngeal carcinoma HIF-1 alpha radiosensitivity** ("larynges"[All Fields] OR "laryngitis"[MeSH Terms] OR "laryngitis"[All Fields] OR "laryngitides"[All Fields] OR "larynx"[MeSH Terms] OR "larynx"[All Fields] OR "laryngeal"[All Fields]) AND ("carcinoma"[MeSH Terms] OR "carcinoma"[All Fields] OR "carcinomas"[All Fields] OR "carcinoma s"[All Fields]) AND ("hif1a protein human"[Supplementary Concept] OR "hif1a protein human"[All Fields] OR "hif 1 alpha"[All Fields]) AND ("radiation tolerance"[MeSH Terms] OR ("radiation"[All Fields] AND "tolerance"[All Fields]) OR "radiation tolerance"[All Fields] OR "radiosensitivities"[All Fields] OR "radiosensitivity"[All Fields] OR "radiation sensitizing agents"[Pharmacological Action] OR "radiation sensitizing agents"[Supplementary Concept] OR "radiation sensitizing agents"[All Fields] OR "radiosensitizer"[All Fields] OR "radiation sensitizing agents"[MeSH Terms] OR ("radiation sensitizing"[All Fields] AND "agents"[All Fields]) OR "radiosensitizers"[All Fields] OR "radiosensitisation"[All Fields] OR "radiosensitise"[All Fields] OR "radiosensitised"[All Fields] OR "radiosensitiser"[All Fields] OR "radiosensitisers"[All Fields] OR "radiosensitises"[All Fields] OR "radiosensitising"[All Fields] OR "radiosensitive"[All Fields] OR "radiosensitization"[All Fields] OR "radiosensitize"[All Fields] OR "radiosensitized"[All Fields] OR "radiosensitizes"[All Fields] OR "radiosensitizing"[All Fields]) **Filters/limits: None**
PubMed	**Search: head and neck cancer plasma hypoxia markers** ("head and neck neoplasms"[MeSH Terms] OR ("head"[All Fields] AND "neck"[All Fields] AND "neoplasms"[All Fields]) OR "head and neck neoplasms"[All Fields] OR ("head"[All Fields] AND "neck"[All Fields] AND "cancer"[All Fields]) OR "head and neck cancer"[All Fields]) AND ("plasma"[MeSH Terms] OR "plasma"[All Fields] OR "plasmas"[All Fields] OR "plasma s"[All Fields]) AND ("hypoxia"[MeSH Terms] OR "hypoxia"[All Fields] OR "hypoxia s"[All Fields] OR "hypoxias"[All Fields]) AND ("marker"[All Fields] OR "markers"[All Fields]) **Filters/limits: None**

Supplementary Materials

PMC	**Search: laryngeal carcinoma hypoxia** ("larynges"[All Fields] OR "laryngitis"[MeSH Terms] OR "laryngitis"[All Fields] OR "laryngitides"[All Fields] OR "larynx"[MeSH Terms] OR "larynx"[All Fields] OR "laryngeal"[All Fields]) AND ("carcinoma"[MeSH Terms] OR "carcinoma"[All Fields] OR "carcinomas"[All Fields] OR "carcinoma s"[All Fields]) AND ("hypoxia"[MeSH Terms] OR "hypoxia"[All Fields] OR "hypoxia s"[All Fields] OR "hypoxias"[All Fields]) **Filters/limits: None**
PMC	**Search: laryngeal carcinoma HIF-1 alpha** ("larynges"[All Fields] OR "laryngitis"[MeSH Terms] OR "laryngitis"[All Fields] OR "laryngitides"[All Fields] OR "larynx"[MeSH Terms] OR "larynx"[All Fields] OR "laryngeal"[All Fields]) AND ("carcinoma"[MeSH Terms] OR "carcinoma"[All Fields] OR "carcinomas"[All Fields] OR "carcinoma s"[All Fields]) AND ("hif1a protein human"[Supplementary Concept] OR "hif1a protein human"[All Fields] OR "hif 1 alpha"[All Fields]) **Filters/limits: None**
PMC	**Search: laryngeal carcinoma VEGF** ("larynges"[All Fields] OR "laryngitis"[MeSH Terms] OR "laryngitis"[All Fields] OR "laryngitides"[All Fields] OR "larynx"[MeSH Terms] OR "larynx"[All Fields] OR "laryngeal"[All Fields]) AND ("carcinoma"[MeSH Terms] OR "carcinoma"[All Fields] OR "carcinomas"[All Fields] OR "carcinoma s"[All Fields]) AND ("vascular endothelial growth factor a"[Supplementary Concept] OR "vascular endothelial growth factor a"[All Fields] OR "vegf"[All Fields] OR "vascular endothelial growth factor a"[MeSH Terms]) **Filters/limits: None**
PMC	**Search: laryngeal carcinoma GLUT1** ("larynges"[All Fields] OR "laryngitis"[MeSH Terms] OR "laryngitis"[All Fields] OR "laryngitides"[All Fields] OR "larynx"[MeSH Terms] OR "larynx"[All Fields] OR "laryngeal"[All Fields]) AND ("carcinoma"[MeSH Terms] OR "carcinoma"[All Fields] OR "carcinomas"[All Fields] OR "carcinoma s"[All Fields]) AND "GLUT1"[All Fields] **Filters/limits: None**
